# Venoarterial extracorporeal membrane oxygenation for cardiac support in human immunodeficiency virus-positive patients: a case report and review of a multicentre registry

**DOI:** 10.1186/s13019-023-02191-8

**Published:** 2023-04-07

**Authors:** Matthew Laraghy, James McCullough, John Gerrard, Andrie Stroebel, James Winearls

**Affiliations:** 1 Intensive Care, Gold Coast University Hospital, Gold Coast, QLD Australia; 2 Infectious Diseases and Immunology, Gold Coast University Hospital, Gold Coast, QLD Australia; 3 Cardiothoracic Surgery, Gold Coast University Hospital, Gold Coast, QLD Australia; 4 School of Medicine, University of Queensland, St. Lucia, QLD Australia; 5 School of Public Health and Preventive Medicine, Monash University, Melbourne, VIC Australia; 6 St Andrew’s War Memorial Hospital, Brisbane, QLD Australia

**Keywords:** Cardiogenic shock, Extracorporeal life support (ECLS), Extracorporeal life support Organization (ELSO), Extracorporeal membrane oxygenation (ECMO), Human immunodeficiency virus (HIV), Venoarterial

## Abstract

**Background:**

Human immunodeficiency virus (HIV) is associated with increased risk of heart failure via multiple mechanisms both in patients with and without access to highly active antiretroviral therapy (HAART). Limited information is available on outcomes among this population supported on Venoarterial Extracorporeal Membrane Oxygenation (VA ECMO), a form of temporary mechanical circulatory support.

**Methods:**

We aimed to assess outcomes and complications among patients with HIV supported on VA ECMO reported to a multicentre registry and present a case report of a 32 year old male requiring VA ECMO for cardiogenic shock as a consequence of his untreated HIV and acquired immune deficiency syndrome (AIDS). A retrospective analysis of the Extracorporeal Life Support Organization (ELSO) registry data from 1989 to 2019 was performed in HIV patients supported on VA ECMO.

**Results:**

36 HIV positive patients were reported to the ELSO Database who received VA ECMO during the study period with known outcomes. 15 patients (41%) survived to discharge. No significant differences existed between survivors and non-survivors in demographic variables, duration of VA ECMO support or cardiac parameters. Inotrope and/or vasopressor requirement prior to or during VA ECMO support was associated with increased mortality. Survivors were more likely to develop circuit thrombosis. The patient presented was supported on VA ECMO for 14 days and was discharged from hospital day 85.

**Conclusions:**

A limited number of patients with HIV have been supported with VA ECMO and more data is required to ascertain the indications for ECMO in this population. HIV should not be considered an absolute contraindication to VA ECMO as they may have comparable outcomes to other patient groups requiring VA ECMO support.

## Introduction

Human immunodeficiency virus (HIV) infection is associated with an increased incidence of cardiac disease including coronary artery disease, cardiomyopathy, valvular disease, pericardial disease and pulmonary hypertension [1, 2]. This increased risk is conferred via multiple mechanisms, including direct effects of HIV, abnormal inflammatory processes, opportunistic infections, nutritional deficiencies and drug toxicities leading to structural and arrhythmic complications [1].

Initial presentation with HIV varies depending on access to highly active antiretroviral therapy (HAART) [3]. This has resulted in the rate of HIV progression to acquired immune deficiency syndrome (AIDS), which previously was the main cause of mortality and morbidity, to be reduced in high income countries (HIC) [4]. Treated HIV is now viewed as a chronic disease with 25% of the deaths in the post HAART- era due to cardiac disease in HIC [2].

Previous studies have shown there is unequal access to advanced cardiac failure treatments for HIV patients. Reasons for this disparity include resource limitations, lack of experience in this patient population or clinician opinion of their immunocompromised status [5]. Despite this, Extracorporeal Membrane Oxygenation (ECMO), a recognised salvage therapy for cardiac and respiratory failure, has been implemented in HIV AIDS patients for both respiratory and cardiac failure [6–9]. A previous multicentre review of 126 patients supported on ECMO reported a mortality of 64% and a Venoarterial (VA) ECMO mortality of 74% in HIV positive patients [10]. There have been limited case reports of successful VA ECMO support treatment [11, 12].

We present the case of a 32 year old male requiring V-A ECMO for cardiogenic shock as a consequence of his untreated HIV AIDS along with a retrospective review of the Extracorporeal Life Support Organization (ELSO) Registry for VA ECMO.

## Methods

### ELSO registry data

The Extracorporeal Life Support Organization (ELSO) registry collects data on ECMO used to support cardiorespiratory function. Data submitted to the registry is supplied by a standardised ELSO form from respective institutions. Data is limited to the hospitalisation that included ECMO support. The decision to instigate ECMO is institution dependent. Approval for this analysis and report was granted by the Registry Committee of ELSO. De-identified data was released on all patients who received VA ECMO and had an ICD9 or ICD10 primary or secondary diagnosis for HIV between 1989 and 2019. Extracorporeal Cardiopulmonary Resuscitation (ECPR) patients were excluded. A total of 38 patients met these criteria from a pool of 93,901 adults and children from 614 ECMO units. Two patients remained on ECMO at time of analysis and were excluded from the study. All patients had only one exposure to ECMO.

### Statistical analysis

Demographics, diagnosis, Pre-ECMO and ECMO support details and ECMO complications were compared between patients who survived to discharged and non-survivors. Categorical data was compared using a Fisher’s exact test. Most variables were not normally distributed and a Mann U-Whitney test was completed for all continuous variables.

### Case report

Written informed consent was obtained from the patient. The research proposal collection of data was reviewed by the Chair of the Gold Coast Hospital and Health Service Human Research Ethics Committee.

## Case report

A 32-year-old male presented to his primary care physician in September 2019 due to subjective fevers, dysphagia, non-productive cough, back pain, weight loss and faecal incontinence. He had moved to Australia two years prior to presentation from Southeast Asia and reported no previous medical or surgical history.

On initial review he was cachectic, febrile, hypoxic, tachycardic and hypotensive. On examination he had weak peripheral pulses, no murmurs and diminished breath sounds on his left side. Neurological assessment found him Glasgow Coma Scale 15 with a left sided facial droop and upper limb weakness was observed. The abdominal exam was unremarkable.

Clinical and radiological examination revealed a large left sided pneumothorax, which was decompressed with an intercostal catheter. Computer tomography (CT) brain revealed multifocal hypodense lesions. Pathology on admission revealed a haemoglobin (Hb) of 83 g/L and lymphocyte count of 0.46 × 10^9^/L.

He was admitted to the intensive care unit, initiated on broad spectrum antibiotics and fluid resuscitated. Corticosteroids and Trimethoprim/Sulfamethoxazole were commenced due to clinical suspicion of an immunocompromised state with lymphopenia and bilateral lung infiltrate potentially consistent with *Pneumocystis jirovecii pneumonia* (PJP). Initially he was trialled on non-invasive ventilation, however he remained in hypoxic respiratory failure necessitating intubation and vasopressor support. On Day 2 (D2) of hospital admission, a beside transthoracic echocardiograph (TTE) revealed biventricular failure with ejection fraction of 10%, with no valvular pathology and a small/moderate anterior pericardial effusion. He was transferred to the regional ECMO centre. On arrival arterial blood gas showed a pH 7.17, pCO2 33mmHg, pO2 128mmHg, HCO3 12mmol/L, Lactate 6.3mmol/L with an A-a gradient of 400mmHg.

A multidisciplinary team decided to place the patient on V-A ECMO as a bridge to diagnosis as the precipitant for his presentation was unknown at this time and his apparent good premorbid status. While there was a clinical suspicion of HIV with PJP treatment commenced on admission, HIV status was not known prior to ECMO initiation.

V-A ECMO was established percutaneously with ultrasound and transesophageal echocardiographic guidance in a V21_f_-A17_f_d8_t_ configuration. The left femoral artery was cannulated with a 17 French (Fr) arterial return catheter, and an 8 Fr antegrade distal perfusion catheter was placed in the superficial femoral artery. The right femoral vein was cannulated with a multistage 21 Fr venous access catheter. Initial V-A ECMO blood flow was 3 L/min and sweep gas flow 3 L/min with FsO2 100%. Ventilatory settings were adjusted to a respiratory rate of 10 breaths per minute, FiO2 60%, positive end-expiratory pressure (PEEP) 8cmH20 and Pressure Support 12cmH20. He was anticoagulated on a heparin infusion targeting an activated partial thromboplastin time (APTT) of 70–90 s to limit patient and circuit thrombosis. To ensure the target range was reached, APTT was tested every 6 hours. Daily coagulation studies (APTT, prothrombin time and fibrinogen levels), anti-Xa level, antithrombin III level, plasma free haemoglobin and rotational thromboelastometry (ROTEM) were also performed.

Once stabilised on V-A ECMO, investigations included high resolution CT revealing extensive bilateral pulmonary consolidation, multiple liver abscesses, a right retroperitoneal abscess, cavitating left apical lung lesion and focal destruction of the T11 vertebral body.

He was found to be HIV antibody positive and immunosuppressed with CD4 count = 0.02 × 10^9^/L. Results of viral load testing can be seen in Table 1. Clinical diagnoses of PJP and cerebral *toxoplasmosis* were subsequently confirmed by polymerase chain reaction (PCR) performed on bronchoalveolar lavage fluid and cerebral spinal fluid respectively. Appropriate antimicrobial agents were initiated under the guidance of the infectious diseases team. He was started on HAART D3.

**Table 1:** Results of Viral Load Tests and Lymphocyte Testing

	Hospital day		
Variable	1	28	57
HIV-1 RNA PCR (copies/ml)	75,800	56,700	3,840
HIV-1 RNA PCR log10* ( Log copies/ml)	4.9	4.8	3.6
CD4 absolute count (0.56–1.58 × 10^9^/L)	0	0	0.02
CD8 absolute count (0.25–0.90 × 10^9^/L)	0	0	0.14

* Result obtained using the Roche cobas 6800 HIV−1 Quantitative assay.

Additional pathology revealed a Ferritin of 91,600 μg/L and a Lactate Dehydrogenase of 1900 U/L. Due to clinical suspicion for potential Hemophagocytic Lymphohistiocytosis (HLH) a bone marrow aspirate was performed, and the patient was empirically treated with dexamethasone 20 mg D2-4. Histology showed reactive features with no evidence of HLH and a weaning regime of hydrocortisone was commenced. A cardiac muscle biopsy was not performed during his intensive care or hospital admission.

Due to acute respiratory distress syndrome (ARDS) and haemodynamic compromise the patient was paralysed on a cisatracurium infusion day D2-6 and inhaled nitric oxide was instituted D2-5.

An infective aetiology of his multiple liver lesions was suspected, and diagnostic ultrasound guided aspiration was performed D3 with two pigtail drains inserted. The patient was on a heparin infusion at 7 units/kg/hr with an APTT of 35 s checked prior to procedure. The infusion was continued as the risk of circuit thrombosis was thought to exceed the risk of haemorrhage secondary to the pigtail insertion. Unfortunately, the procedure was complicated by frank bleeding from drains requiring multiple transfusions and point-of-care ROTEM guided product replacement. CT angiogram did not demonstrate a culprit arterial bleed and it was presumed of venous origin in the setting of anticoagulation for ECMO. Haemostasis was attempted with instillation of Gelfoam through the drains into the abscess cavity. On D4 it appeared the bleeding had ceased as the Hb was stable and the patient did not have an ongoing transfusion requirement. The heparin infusion was continued throughout this period however APTT target levels were reduced to 60–80 s. On D5 a drain was removed and bleeding was noted at the insertion site. Subsequently the patient developed intra-abdominal hypertension with abdominal pressures measured at 40 cmH20 on D9. This impaired ventilation and contributed to circuit access insufficiency. A further CT angiogram was performed, which did not reveal a source of bleeding. The heparin infusion was ceased and an ultrasound guided abdominal aspiration drained 3 L frank blood with clots. Heparin was recommenced at 5 units/kg/hr 4 hours post abdominal pigtail insertion and bleeding ceased on D10. The second liver drain was removed D14. There were no circuit issues despite non-therapeutic anticoagulation and ECMO ΔP values remained static at 13-15mmHg until time of decannulation. No pathogens were cultured from the liver aspirates.

Serial TTE showed improved cardiac function with left ventricle ejection fraction (LVEF) improving to 20% and decannulation from V-A ECMO occurred on D14. Over the course of ICU admission, the patient developed a severe acute kidney injury (AKI) requiring regular renal replacement therapy D4-14. Post ECMO decannulation he responded to a frusemide challenge with resolution of AKI.

His care was further complicated by a left pleural effusion. Pleural fluid cultured *Candida albicans* which was treated with fluconazole. He had a prolonged ventilatory wean due to critical illness myopathy and was extubated D30. Neurological assessment post extubation revealed bilateral vocal cord palsy. He was discharged from the ICU and was engaged in rehabilitation on D38. He was discharged from hospital D85 and further follow up 8 months after presentation he was well in the community on HAART.

## ELSO database results

A total of 36 patients with known outcomes received ECMO support with a diagnosis code of HIV between 1989 and 2019. 15 patients (41%) survived to discharge. Median age was 39 (interquartile range (IQR): 27–55) years. Median weight was 67 (IQR: 59–89) Kg. No significant differences existed between survivors and non-survivors in baseline demographic variables (Table 2). Primary diagnoses are summarised in Table 3. Pre-ECMO inotrope and/or vasopressor support was associated with increased mortality (p = 0.002). No other Pre-ECMO adjunctive treatment was associated with poor outcomes (Table 2). Inotrope and/or vasopressor requirement on ECMO was significantly higher in non-survivors than survivors (p = 0.02) while circuit component clots occurred more often in the survivor group (p = 0.01) (Table 4). Otherwise, there was no significant difference between other complications. Non- Survivors remained on ECMO for a median of 93 (IQR 38.5–157) hours while survivors remained on ECMO for 137 h (IQR: 64–200 h) (p = 0.18).

**Table 2:** Baseline Demographic, Pre-VA ECMO Cardiac Parameters and Adjunct Pre-VA ECMO Treatment of Non-Survivors and Survivors

	Non-Survivors (21)	Survivors (15)	*p*-value
Demographics			
Weight (kg), Median (IQR)	65.8 (56–93)	67 (59–88.6)	0.748
Age (years), Median (IQR)	48 (29–56)	32 (24–55)	0.194
Female, n (%)	5 (24)	4 (27)	0.57
Ethnicity, n (%)			
Asian	0 (0)	1 (7)	0.682
Black	13 (62)	9 (60)	
Hispanic	2 (10)	2 (13)	
Unknown	1 (5)	0 (0)	
White	5 (24)	3 (20)	
Pre-ECMO Cardiac Parameters			
Systolic blood pressure (mmHg), median (IQR)	87.5 (73.5–95.25)	86 (68.5–107)	0.884
Diastolic blood pressure (mmHg), median (IQR)	53 (47.5–57.25)	58.5 (52.25–69.25)	0.078
Adjunct Pre-VA ECMO Treatment, n (%)			
Vasopressor/inotropic drugs*	19 (90)	6 (40)	0.0024
Cardiopulmonary bypass	2 (10)	2 (13)	1
Intra-aortic balloon	3 (14)	2 (13)	1
Nitric oxide	2 (10)	1 (7)	1
Neuromuscular blockers	6 29)	1 (7)	0.2
Bicarbonate (Intravenous)	5 (24)	2 (13)	0.67
Renal Replacement Therapy	4 (19)	1 (7)	0.38

*Vasopressor/inotropic drugs include Adrenaline, Noradrenaline, Vasopressin, Milrinone and Levosimendan and Vasoactive drug not specified.

**Table 3:** Primary Diagnosis of Non-Survivors and Survivors

Diagnosis	Non-Survivors (21)	Survivors (15)
Infectious		
Sepsis with or without shock	3	0
Circulatory		
Myocardial Infarction	1	1
Cardiac Arrest	3	1
Cardiogenic Shock	5	4
Mitral Valve Disease	0	1
Infective Endocarditis	0	1
Acute Myocarditis	1	0
Heart Failure	2	0
Post Cardiac Surgery	1	0
Complication of Cardiac Implant	1	0
Ventricular Tachyarrhythmia	0	2
Atrial Arrythmia	1	0
Arrythmia Unspecified	0	1
Pulmonary Embolism	0	2
Pulmonary Hypertension	1	1
Pharmacological		
Beta Blocker Toxicity	0	1
Other		
Post-Surgical shock	1	0
Shock unspecified	1	0

**Table 4:** Complications After Placement on VA ECMO of Non-Survivors and Survivors

	Non-Survivors (21)	Survivors (15)	*p*-value
Cardiovascular, n (%)			
Cardiac arrhythmia	3 (14)	1 (7)	0.6257
CPR	3 (14)	0 (0)	0.25
Vasopressor/Inotropes on VA ECMO	9 (42)	1 (7)	0.0245
Myocardial stunning	3 (14)	0 (0)	0.25
Neurologic, n (%)			
Brain death	2 (10)	0 (0)	0.5
CNS Infarction	1 (5)	0 (0)	1
Pulmonary, n (%)			
Pneumothorax requiring treatment	1 (5)	1 (7)	1
Pulmonary haemorrhage	1 (5)	0 (0)	1
Renal, n (%)			
AKI	7 (33)	1 (7)	0.1038
Renal Replacement Therapy	8 (38)	2 (13)	0.1422
Circuit complications, n (%)			
Thrombosis	1 (5)	6 (40)	0.0134
Other, n (%)			
Haemorrhage	3 (14)	4 (27)	0.4178
Culture proven infection while on VA ECMO	4 (19)	2 (13)	1
Hyperbilirubinemia	3 (14)	0 (0)	0.25

*Vasopressor/inotropic drugs include Adrenaline, Noradrenaline, Vasopressin, Milrinone and Levosimendan and Vasoactive drug not specified.

## Discussion

The causes of cardiac disease in individuals with HIV is influenced by treatment with HAART [1]. This is reflected in the heterogenous aetiology of cardiac failure in our review, evident in the primary diagnoses (Table 3). In the Pre-HAART era HIV-Cardiomyopathy was associated with poor prognosis [2]. Described as a decreased LVEF or dilated LV by imaging studies, with or without symptoms of heart failure, it is a recognised complication of HIV infection [2]. In HIC it is now a rare presentation however untreated and undiagnosed disease can still present acutely [5, 13], as was our patients experience.

With HAART, HIV-cardiomyopathy more commonly presents as diastolic dysfunction [14]. HIV patients are also at increased risk of other causes of cardiac failure, notably coronary artery disease (CAD). With increasing life expectancy and high rates of traditional risk factors, HIV positive individuals are at 1.5 to 2 fold increased risk of CAD [15]. While they have increased incidence of major cardiac event, 30 day and 1 year mortality is comparable to patients without HIV [16]. They also tend to be younger, receive less myocardial infarction related interventions and are more often to be re-hospitalised with ischemic cardiomyopathy [15].

Limited data is available looking at outcomes of cardiac failure in the HIV population requiring VA ECMO. Mortality in this review of the ELSO registry was 59% which is consistent with other studies reporting VA ECMO mortality ranging from 50 to 70% for cardiogenic shock [7, 8, 17–19]. While there was a trend to survivors being younger, this was not statistically significant. Non-Survivors required more inotropic and vasopressor support prior to ECMO and while on support, consistent with previous studies [10].

Support with VA ECMO is associated with significant morbidity and complications, as shown in Table 4. Both bleeding and thrombotic complications were common in the registry data, highlighting the challenges of haemostatic management in these patients. Pre-existing coagulopathy due to acute illness, exposure to the ECMO circuit and requisite anticoagulation contribute to both bleeding and thrombosis risk. HIV infection is recognised as a prothrombotic state [20] due to multiple mechanisms and further research is required to determine its contribution to these complications in this unique setting. No data was provided on anticoagulation, platelet count or coagulation profiles by the ELSO registry.

Our patient reflects the complexity of managing these patients. He experienced multiple complications including haemorrhage, and renal failure. He also required vasopressor and inotropic support, which was associated with poor outcomes in our review of the ELSO registry.

This study had significant limitations. Limited cardiovascular parameters were provided to compare between survivors and non-survivors by ELSO registry. No echocardiographic findings were available for comparison. Data did not stipulate whether patients were on antiretroviral therapy prior to placement on ECMO, or whether the diagnosis of HIV was known. ICU mortality scores, and prognostic indicators specific to HIV individuals, could not be assessed. No data was provided on centre location, patient selection, or cessation of ECMO, therefore institutional influence could not be evaluated. Diagnoses were recorded as ICD-9 and ICD-10 codes, which have limitations [21], and it is unclear which of these preceded presentation or cannulation. This was reflected in patients whose primary diagnosis was cardiac arrest which does not indicate cause. Data collection resulted in some fields being empty at time of analysis. Results must also be taken in context of the small sample size.

The HIV population requiring VA ECMO support for cardiac failure has a varied aetiology, as shown in our case study, the review of ELSO registry and previously published case studies [11, 12, 22, 23]. This limits comparison between cases and the generality of our study’s findings. Therefore, there is significant scope for further research.

The decision to support cardiac failure with VA ECMO is complex. There is a paucity of evidence to guide therapy, it is a labour-intensive therapy and is unproven in many patient populations. Comorbidities further complicate this assessment. Previous studies have shown there is unequal access to care for HIV patients to advanced cardiac failure treatment [5]. While our patient had a good outcome, further studies are needed to further analyse factors which are associated with favourable outcomes to assist with clinical decision making.

## Conclusion

HIV patients with cardiogenic failure have comparable outcomes to other patient populations requiring VA ECMO and therefore should be considered for support. This study highlights the lack of data relating to HIV patients supported on VA ECMO due to the limited application of this therapy in this population. Further studies are required to elucidate which patient factors predict favourable outcomes however HIV diagnosis alone should not preclude treatment with VA ECMO.

## Data Availability

The datasets used and/or analysed during the current study are available from the corresponding author on reasonable request.

## Abbreviations

AIDS: Acquired immune deficiency syndrome
AKI: Acute kidney injury
APTT: Activated partial thromboplastin time
CT: Computer tomography
CAD: Coronary artery disease
ECPR: Extracorporeal cardiopulmonary resuscitation
ECLS: Extracorporeal life support
ELSO: Extracorporeal Life Support Organization
ECMO: Extracorporeal membrane oxygenation
Fr: French
HIC: High income countries
HAART: Highly active antiretroviral therapy
HIV: Human immunodeficiency virus
IQR: Interquartile range
PJP: Pneumocystis jirovecii pneumonia
PEEP: Positive end-expiratory pressure
ROTEM: Rotational thromboelastometry
TTE: Transthoracic echocardiograph
VA: Venoarterial (mode)
V-A: Venoarterial (configuration)
